# Highly-efficient photocatalytic degradation of methylene blue by PoPD-modified TiO_**2**_ nanocomposites due to photosensitization-synergetic effect of TiO_2_ with PoPD

**DOI:** 10.1038/s41598-017-04398-x

**Published:** 2017-06-21

**Authors:** Chuanxi Yang, Wenping Dong, Guanwei Cui, Yingqiang Zhao, Xifeng Shi, Xinyuan Xia, Bo Tang, Weiliang Wang

**Affiliations:** 1 College of Geography and Environment, Shandong Normal University, Jinan, 250014 P. R. China; 2 College of Resources and Environmental Sciences, China Agricultural University, Beijing, 100193 P. R. China; 3 Shandong Academy of Environmental Science and Environmental Engineering Co, Ltd, Jinan, 250013 P. R. China; 4 College of Chemistry, Chemical Engineering and Materials Science, Collaborative Innovation Center of Functionalized Probes for Chemical Imaging in Universities of Shandong, Key Laboratory of Molecular and Nano Probes, Ministry of Education, Shandong Provincial Key Laboratory of Clean Production of Fine Chemicals, Shandong Normal University, Jinan, 250014 P. R. China

## Abstract

Poly-o-phenylenediamine modified TiO_2_ nanocomposites were successfully synthesized via an ‘*in situ*’ oxidative polymerization method. The modified nanocomposites were characterized by BET, XRD, TEM, FT-IR, TGA, XPS, EA and UV-Vis DRS. The photocatalytic degradation of methylene blue was chosen as a model reaction to evaluate the photocatalytic activities of TiO_2_ and PoPD/TiO_2_. The results indicated that PoPD/TiO_2_ nanocomposites exhibited good photocatalytic activity and stability. The photocatalytic activity of PoPD/TiO_2_ increased as the initial pH increased because of electrostatic adsorption between the photocatalyst and MB as well as the generation of ·OH, whereas it exhibited an earlier increasing and later decreasing trend as the concentration of the photocatalyst increased owing to the absorption of visible light. The photocatalytic stability of the PoPD/TiO_2_ nanocomposite was dependent on the stability of its structure. Based on radical trapping experiments and ESR measurements, the origin of oxidizing ability of PoPD/TiO_2_ nanocomposites on photocatalytic degradation of MB was proposed, which taking into account of ·OH and ·O_2_^−^ were the first and second important ROS, respectively. The possible photocatalytic mechanism and photocatalytic activity enhanced mechanism has been proposed, taking into account the photosensitization effect and synergetic effect of TiO_2_ with PoPD.

## Introduction

In the field of environmental chemistry, the use of semiconductors as photocatalysts has been the focus of recent attention since it aims at the destruction of contaminants in water and air^1, 2^. Among the semiconductors, titanium dioxide (TiO_2_) is an excellent photocatalyst because it is an effective, photostable, reusable, inexpensive, non-toxic and easily available catalyst^3–5^. However, the wide band gap (3.2 eV) of TiO_2_ only allows it to absorb ultraviolet light (<387 nm), which limits the utilization of solar light since UV light represents less than 5% of solar light^6, 7^.

To extend the photoresponse of TiO_2_ to the visible region, many modification methods, such as metal ion doping^8, 9^, non-metal doping^10, 11^, noble metal deposition^12^, composite semiconductors^13, 14^ and surface dye sensitization^15, 16^ have been reported. Recently, a large body of work has been produced based on using conjugated polymer-modified TiO_2_ to degrade organic pollutant because nanocomposites of conductive polymers and inorganic particles show interesting physical properties and application potential^17–19^. Some studies have been published on the combination of conductive polymers and TiO_2_ to improve performance under UV light and sunlight conditions. For example, Zhang *et al*.^20^ reported that PANI-modified TiO_2_ nanocomposites showed a higher photocatalytic activity than TiO_2_ under ultraviolet light and visible light, and the enhancement was attributed to the synergetic effect between TiO_2_ and PANI. As a typical conducting polymer, poly-o-phenylenediamine (PoPD) has attracted considerable attention since its discovery^21, 22^. Taking advantage of the unique electrical, optical and photoelectric properties of PoPD, we expect that the combination of PoPD with TiO_2_ may induce an interesting charge transfer and thus enhance the photocatalytic activity of TiO_2_ under visible light irradiation. However, the photocatalytic activity enhanced mechanism has not been studied.

In our studies, PoPD/TiO_2_ nanocomposites were synthesized via an ‘*in situ*’ oxidative polymerization method. The modified photocatalysts were characterized by BET Test, X-ray diffraction (XRD), transmission electron microscopy (TEM), Fourier-transform infrared spectroscopy (FT-IR), thermogravimetric analysis (TGA), X-ray photoelectron spectroscopy (XPS), Elemental Analysis (EA) and UV-Vis diffuse reflectance spectroscopy (UV-Vis DRS). The results indicated that the PoPD exists on the surface of TiO_2_, the presence of PoPD does not impact on the lattice structure and grain size of TiO_2_, and the presence of PoPD enhances the visible light response. The photocatalytic degradation of methylene blue (MB) was chosen as a model reaction to evaluate the photocatalytic activities of TiO_2_ and PoPD/TiO_2_. The results indicated that PoPD/TiO_2_ nanocomposites exhibited good photocatalytic activity (apparent first-order rate constants of 0.0021 min^−1^ for TiO_2_ and 0.0033 min^−1^ for P/T(1/4)) and stability (recycled 5 times, 15 hours of operation). The photocatalytic activity of PoPD/TiO_2_ was influenced by the initial pH and concentration of the photocatalyst. Based on radical trapping experiments and electron spin resonance (ESR) measurements, the origin of oxidizing ability of PoPD/TiO_2_ nanocomposites on photocatalytic degradation of MB was proposed, which taking into account of ·OH and ·O_2_^−^ were the first and second important ROS, respectively. The photocatalytic activity enhanced mechanism has been proposed, taking into account the photosensitization effect (UV-Vis DRS) and synergetic effect of TiO_2_ with PoPD (synergetic factor and electrochemical impedance spectroscopy (EIS)).

## Experimental

### Reagents and materials

TiO_2_ was purchased from Degussa with a BET specific surface area of 291.380 m^2^/g. The o-phenylenediamine (oPD) and ammonium persulfate (APS) were purchased from Tianjin Kermel Chemical Reagent Co., Ltd. Ethyl alcohol was purchased from Tianjin Fuyu Fine Chemical Co., Ltd. Hydrochloric acid was purchased from Sinopharm Chemical Reagent Co., Ltd. Methylene blue was purchased from Tianjin Guangcheng Chemical Reagent Co., Ltd. All of the chemicals were of analytical grade and used without further purification. Deionized water was used for the preparation of all of the solutions.

### Preparation of PoPD/TiO_2_ nanocomposites

The typical synthesis of the PoPD/TiO_2_ nanocomposites is described below.

An appropriate amount of oPD was dissolved in 90 ml of a 1.2 mol/L hydrochloric acid solution followed by the addition of 0.512 g of TiO_2_. The solution was ultrasonicated for 15 min to ensure uniform mixing. After dissolution, the solution was labeled A. An appropriate amount of APS was dissolved in 30 ml of a 1.2 mol/L hydrochloric acid solution, and this solution was labeled B. After solution A was transferred to a 250 ml round-bottom flask, a magneton was added, and the solution was stirred with a magnetic stirrer. Then, solution B was transferred to a 100 mL constant pressure funnel, and solution A was added dropwise at approximately 1 drop/second with stirring. The reaction was continued for 24 hours at room temperature. The final products were filtered and washed with deionized water and ethanol followed by drying at 80 °C for several hours in a vacuum oven. In the experiment, different initial molar ratios of oPD to TiO_2_ (from 1/6 to 4/1) were employed to obtain TiO_2_ nanocomposites deposited by PoPD. In this fashion, a series of PoPD/TiO_2_ nanocomposites with various initial molar ratios of oPD to TiO_2_ (i.e., 1/6, 1/5, 1/4, 1/2, 1/1, 2/1, 3/1, and 4/1) were prepared, and these nanocomposites are referred to as P/T(1/6), P/T(1/5), P/T(1/4), P/T(1/2), P/T(1/1), P/T(2/1) P/T(3/1), and P/T(4/1), respectively. To confirm the effect of PoPD in the composites, the TiO_2_ nanocomposites were treated with the same procedure as that used for the composites without the addition of oPD.

### Characterizations of PoPD/TiO_2_ nanocomposites

The surface texture of TiO_2_ and PoPD/TiO_2_ nanocomposite was examined by N_2_ adsorption at 77 K (Quantachrome instruments Quadrasorb SI). The specific surface area was calculated from the N_2_ adsorption isotherm using the BET equation. X-ray diffraction (XRD) patterns were recorded on a Bruker D8 Advance X-ray diffractometer with Cu Kα radiation. Transmission electron microscopy (TEM) was performed on a JEM-2100 transmission electron microscope. The Fourier-transform infrared spectra (FT-IR) of the samples were recorded on Vertex 70 spectrometer in a range from 4000 to 400 cm^−1^. Thermogravimetric analyses (TGA) of all of the samples were performed with a Q500 thermal analysis instrument. The samples were heated from 35 to 800 °C at a rate of 10 °C min^−1^ in air. The X-ray photoelectron spectroscopy (XPS) measurements were performed using a Thermo ESCALAB 250Xi system with an Al Kα X-ray source. All of the binding energies were referenced to the C1s peak at 284.8 eV for the surface adventitious carbon. Elemental Analysis (EA) was performed on a vario MACRO cube elemental analyzer. Ultraviolet-visible diffuse reflectance spectroscopy (UV-Vis DRS) was performed using a UV-2550PC ultraviolet and visible spectrophotometer from 200 to 800 nm with BaSO_4_ as the background. Electrochemical impedance spectroscopy (EIS) was s performed on a CHI660D VersaSTAT. TiO_2_ and PoPD/TiO_2_ nanocomposites were deposited as a film on a 1 cm × 1 cm indium-tin-oxide conducting glass to obtain the working electrode. The saturated calomel electrode and a Pt electrode served as the reference and counter electrodes, respectively. The electrolyte was 0.1 mol L^−1^ NaClO_4_ solution.

### Photocatalytic activity test

The photocatalytic activities of the samples were evaluated based on the degradation of MB in an aqueous solution under a 1000 W xenon lamp (BL-GHX-V photochemical reactions instrument). Aqueous suspensions of MB (30 ml, 40 mg/L) were placed in a quartz tube, and 30 mg of the PoPD/TiO_2_ nanocomposites were added. Prior to irradiation, the suspensions were magnetically stirred in the dark for approximately 1 h. The suspensions were maintained under constant air-equilibrated conditions before and during illumination. At certain time intervals, 1 ml of the liquor was sampled and centrifuged to remove the particles. The filtrates were analyzed by recording variations in the maximum absorption band (664 nm for MB) using a UV-2550PC ultraviolet and visible spectrophotometer. This process was repeated five times to confirm the stability of the nanocomposites.

## Results and Discussion

### Characterization results of PoPD/TiO_2_ nanocomposites

**Figure 1:**
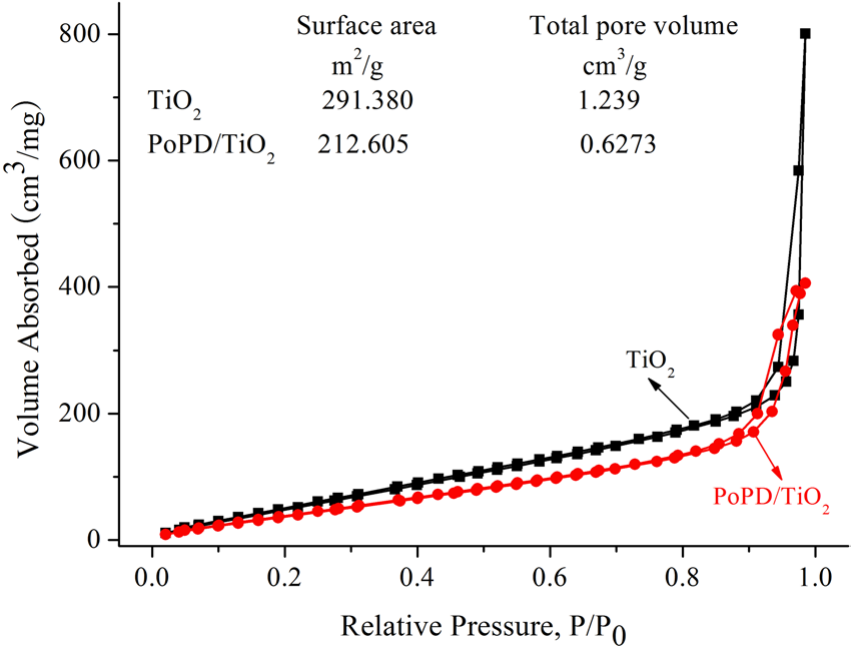
N_2_ adsorption and desorption isotherms at 77 K onTiO_2_ and P/T(1/4).

It was well-known that the BET surface area of sample was an essential parameter for enhanced photocatalytic activity. The adsorption and desorption isotherms of N_2_ at 77 K on TiO_2_ and P/T(1/4) nanocomposites are shown in Fig. 1. Like the P25 TiO_2_, the P/T(1/4) nanocomposite also displays a Type II isotherm characteristic of a mesoporous material. Clearly the total pore volume and surface area of PoPD/TiO_2_ nanocomposite are much less than those of TiO_2_. However, the PoPD/TiO_2_ nanocomposite showed the higher photocatalytic activity than TiO_2_, indicating that the surface area of photocatalyst is only an index to character the physicochemical properties, not the decisive index to ensure the photocatalytic activity.

**Figure 2:**
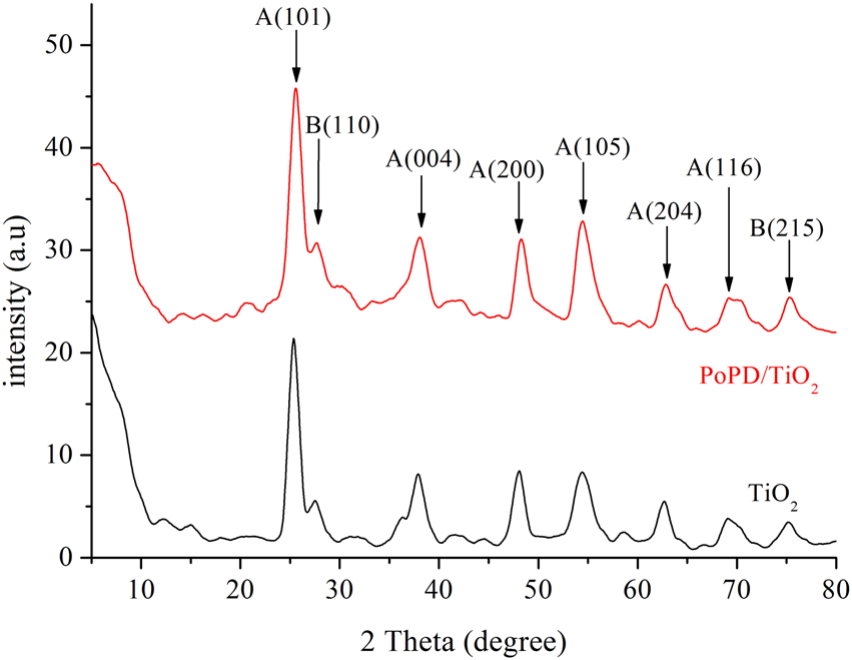
XRD patterns of (a) TiO_2_ and (b) P/T(1/4). (A and B represent anatase and rutile TiO_2_, respectively).

The XRD patterns of TiO_2_ and P/T(1/4) are compared in Fig. 2. The peaks at 2*θ* values of 25.4°, 37.9°, 48.2°, 54.0°, 62.8° and 68.8° can be indexed to the (101), (004), (200), (105), (204) and (116) faces of anatase TiO_2_, respectively. In addition, the peaks at 2*θ* values of 27.5° and 77.3° can be indexed to the (110) and (215) faces of rutile TiO_2_. The peak positions and shapes of the P/T(1/4) nanocomposite did not change compared to those of TiO_2_, indicating that the presence of PoPD does not affect the lattice structure of TiO_2_^23^.

**Figure 3:**
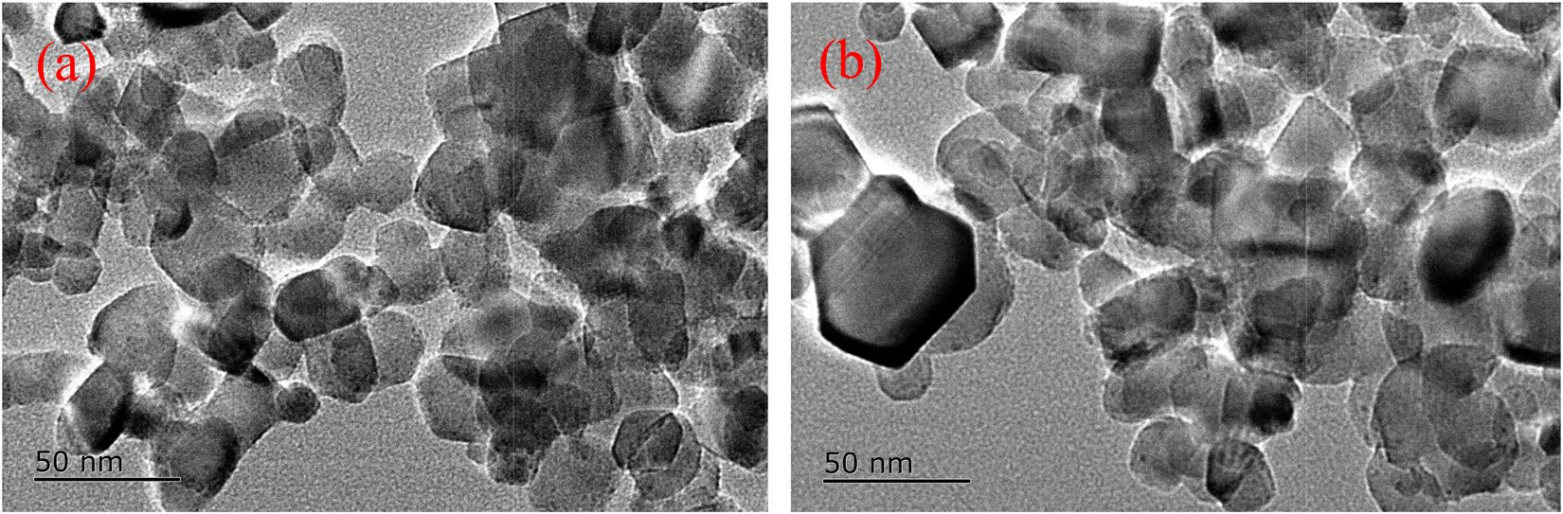
TEM images of (**a**) TiO_2_ and (**b**) P/T(1/4).

The TEM images of TiO_2_ and P/T(1/4) nanoparticles are clearly displayed in Fig. 3(a) and (b). It can be confirmed that the morphology of the P/T(1/4) nanocomposite is similar to that of TiO_2_. In addition, the modification of PoPD does not significantly change the grain size of TiO_2_. The mean sizes of both nanocomposites were approximately 30–50 nm.

**Figure 4:**
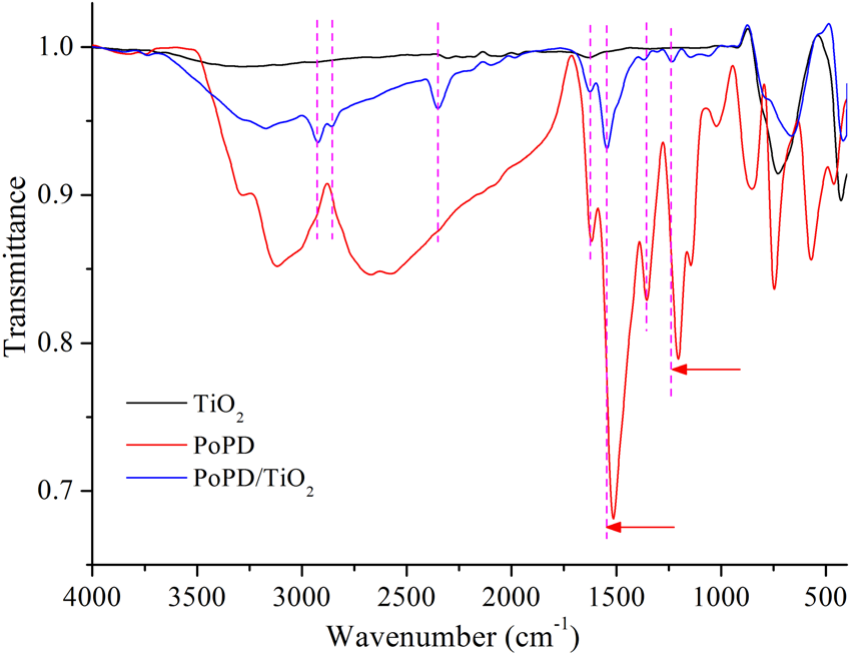
FT-IR spectra of TiO_2_, PoPD, and P/T(1/4).

The FT-IR spectra of TiO_2_, PoPD, and P/T(1/4) are shown in Fig. 4. The main characteristic bands of PoPD are assigned as follows: the intensive peaks between 3500 cm^−1^ and 3200 cm^−1^ can be attributed to the N-H stretching vibrations of the -NH_2_ and -NH- groups. The peak at 1629 cm^−1^ is associated with C=N stretching vibration, and the strong absorption band at 1523 cm^−1^ is ascribed to the C=C stretching vibrations in the benzene ring. The weak peaks at 1328 cm^−1^ and 1238 cm^−1^ are correspondingly assigned to the =C-N stretching on the benzene ring^24^. The FT-IR spectrum of the P/T(1/4) contains the same main characteristic bands as that of PoPD but with a shift to higher wavenumbers^25^. The results show that there is a strong interaction between PoPD and the TiO_2_ nanoparticles, and the PoPD deposits and forms a shell on the surface of the TiO_2_ nanoparticles (2350 cm^−1^, 2850 cm^−1^, and 2925 cm^−1^). The deposition of PoPD on the surface of the TiO_2_ nanoparticles not only constrains the motion of the PoPD chains but also restricts the vibration mode in the PoPD molecule. It can be observed that the characteristic band of TiO_2_ near 500 cm^−1^ occurred in the PoPD/TiO_2_ nanocomposite and the band is so wide that it hides the figure peak in the PoPD/TiO_2_ nanocomposite.

**Figure 5:**
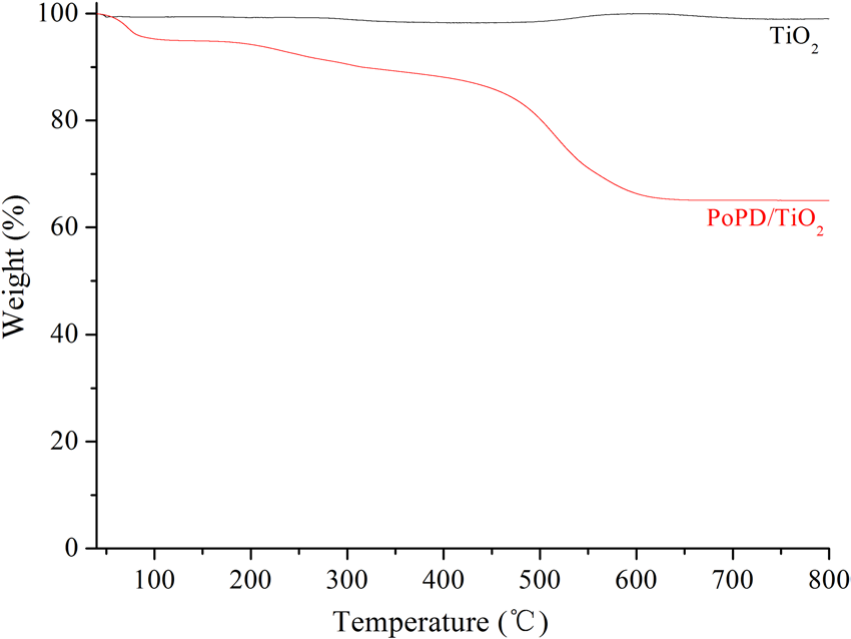
TGA curves of TiO_2_ and P/T(1/4).

The thermal behavior of TiO_2_ and P/T(1/4) was investigated by TGA, and the results are shown in Fig. 5. In Fig. 5, black curve indicates that TiO_2_ is very stable in air, and no decomposition occurred in the 30–800 °C range, and red curve indicates that P/T(1/4) has different showing. The first weight loss was observed at 80 °C owing to desorption of the water that was absorbed on the PoPD. This curve also indicates that a sharp weight loss occurs at approximately 450 °C and continues up to 550 °C. This weight loss was due to decomposition of the skeletal PoPD chain structure^26^.

**Figure 6:**
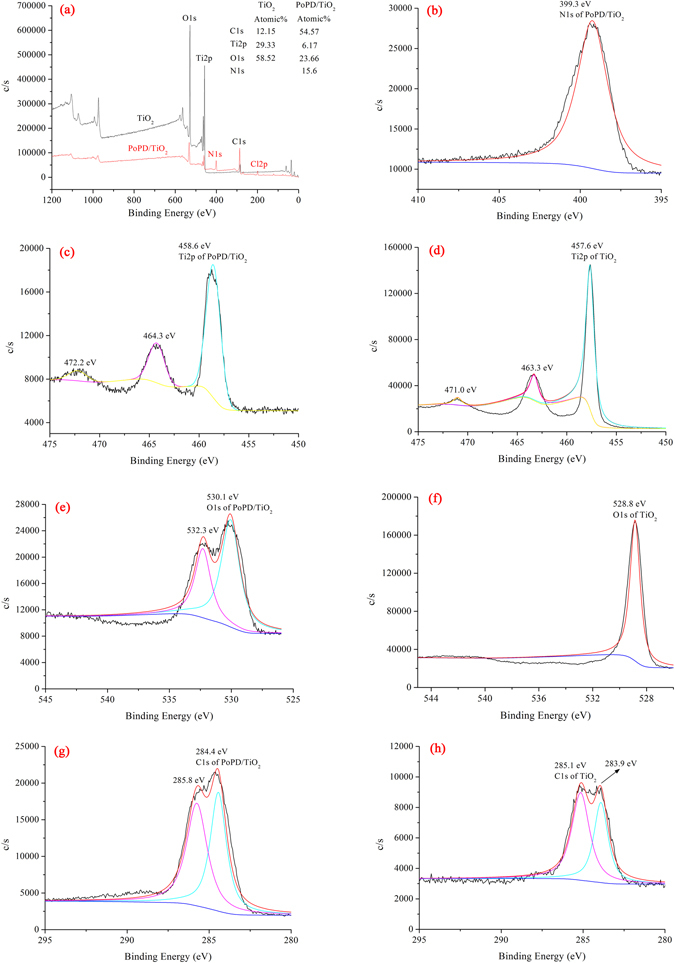
XPS spectra of TiO_2_ and P/T(1/4).

X-ray photoelectron spectroscopy (XPS) is an important tool for studying the electronic structure of condensed matter and is widely used for quantitative surface analysis. According to the XPS survey spectra of TiO_2_ and P/T(1/4), as showing in Fig. 6(a–h), Ti and O were present in TiO_2_ based on the two peaks at binding energies of 458.5 and 529.8 eV. In addition, the C, O, Ti and N elements existed in the P/T(1/4) based on the four peaks with binding energies of 284.8, 529.8, 458.5 and 400.3 eV, which are related to C1s, O1s, Ti2p and N1s, respectively^27^. The atomic percentages of C, O, Ti and N were 54.57%, 23.66%, 6.17% and 15.6%, respectively, suggesting that PoPD exists on the TiO_2_ surface^28^. Meanwhile, as the Fig. 6(e) and 6(f) showing the O1s, it was obviously that there was one peak on O1s of TiO_2_ at 528.8 eV (Ti-O), but there were two peaks on O1s of P/T(1/4) at 530.1 eV (Ti-O) and 532.3 eV (O-PoPD), the results indicated that the PoPD existed on the surface of TiO_2_ and there was an interaction between TiO_2_ and PoPD. The same results were also obtained via Elemental Analysis as Table 1 showing.

**Table 1:** Elemental Analysis results of samples with different initial molar ratios of oPD to TiO_2_.

	volume ratio of N (exceeding rate)	volume ratio of C (exceeding rate)	volume ratio of H (exceeding rate)
blank	0.0435	0.0783	0.13
P/T(1/5)	0.4343 (9.98)	1.7453 (22.29)	0.324 (2.49)
P/T(1/4)	0.4476 (10.29)	1.5825 (20.21)	0.276 (2.12)
P/T(1/2)	0.542 (12.46)	2.0383 (26.03)	0.31 (2.38)
P/T(2/1)	8.5192 (195.84)	26.7795 (342.01)	2.603 (20.02)

**Figure 7:**
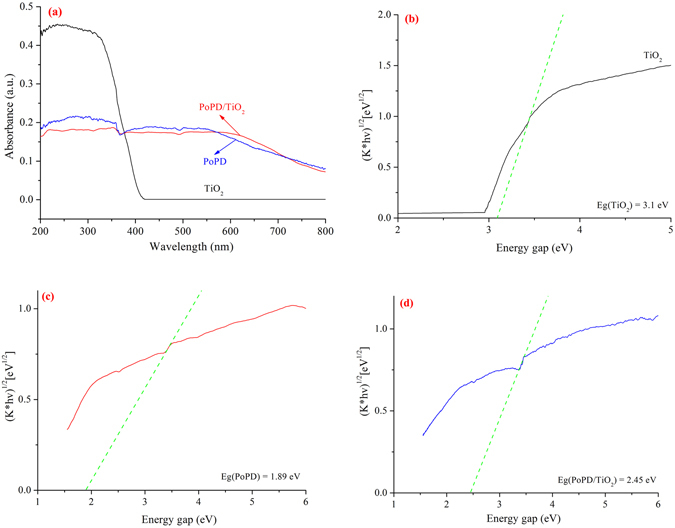
(**a**) UV-Vis DRS spectra of samples and energy gap of (**b**) TiO_2_, (**c**) PoPD, and (**d**) P/T(1/4).

The UV-Vis diffuse reflectance spectra (UV-Vis DRS) of TiO_2_, PoPD and P/T(1/4) are shown in Fig. 7(a). The absorption of both ultraviolet light and visible light by PoPD is similar to that of PoPD/TiO_2_ nanocomposites. In comparison to TiO_2_, the absorption of the P/T(1/4) nanocomposite increases over the entire visible light range but decreases in the UV range. The results indicate that our method is effective for extending the absorption of TiO_2_ to the visible light range^29^. As shown in Fig. 7(b–d), the band gap energies (*E*_*g*_) of TiO_2_, PoPD and PoPD/TiO_2_ nanocomposites, which were obtained from the wavelength values corresponding to the intersection point of the vertical and horizontal portions of the spectra using *hc/λ* = *E*_*g*_, where *E*_*g*_ is the band gap energy, *h* is Planck’s constant, *c* is the speed of light (m/s), and *λ* is the wavelength (nm), were determined to be 3.1 eV, 1.89 eV and 2.45 eV, respectively. Therefore, the PoPD/TiO_2_ nanocomposites can be excited to produce more electron-hole pairs under visible light illumination, which may result in higher photocatalytic activities.

### Photocatalytic activity and stability

**Figure 8:**
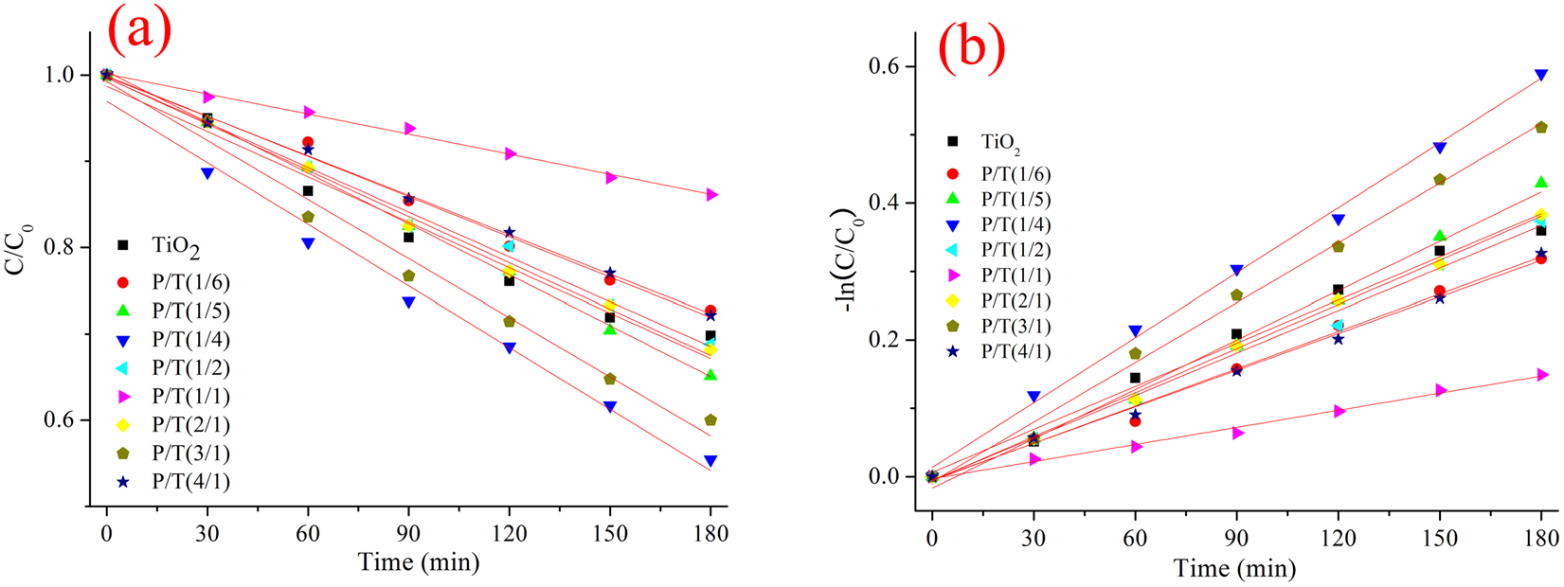
(**a**) The decolorization ratio of MB and (**b**) the apparent first-order linear transforms in the presence of TiO_2_ and PoPD/TiO_2_.

The photocatalytic activity was investigated based on the degradation of MB in an aqueous solution under 1000 W xenon lamp irradiation. MB has a maximum absorption of approximately 664 nm. Figure 8(a) shows the degradation of MB in the presence of TiO_2_ and PoPD/TiO_2_ with different initial ratios of oPD to TiO_2_. The kinetics plots are shown as the apparent first-order linear transform -ln*(C/C*_*0*_ *)* = *k*_*app*_ *t* in Fig. 8(b). The activity of the TiO_2_ and PoPD/TiO_2_ photocatalysts can be evaluated by comparing the apparent first-order rate constants (*k*_*app*_) shown in Table 2. The TiO_2_ and P/T(1/4) nanocomposites have apparent rate constants of 0.0021 min^−1^ and 0.0033 min^−1^, respectively. However, not all of the PoPD/TiO_2_ nanocomposites exhibited higher photocatalytic activity, and an optimal molar ratio between oPD and TiO_2_ exists. P/T(1/4) can obviously enhance photocatalytic activity. The degradation rate of MB exhibited an up-down-up-down trend as the initial molar ratios of oPD to TiO_2_ changed from 1/6 to 4/1. Therefore, the photocatalytic activity was influenced by at least two key factors including solar absorption and charge separation.

**Table 2:** Apparent first-order rate constants (*k*_*app*_) of MB degradation and linear regression coefficients from a plot of -ln(*C/C*_*0*_) = *k*_*app*_ *t*.

Photocatalysts	-ln(*C/C*_*0*_) = *k*_*app*_ *t*	k_app_ (min^−1^)	R^2^
TiO_2_	-ln(*C/C*_*0*_) = 0.0021*t*	0.0021	0.9851
P/T(1/6)	-ln(*C/C*_*0*_) = 0.0018*t*	0.0018	0.9909
P/T(1/5)	-ln(*C/C*_*0*_) = 0.0023*t*	0.0023	0.9895
P/T(1/4)	-ln(*C/C*_*0*_) = 0.0033*t*	0.0033	0.9953
P/T(1/2)	-ln(*C/C*_*0*_) = 0.0020*t*	0.0020	0.9927
P/T(1/1)	-ln(*C/C*_*0*_) = 0.0008*t*	0.0008	0.9916
P/T(2/1)	-ln(*C/C*_*0*_) = 0.0021*t*	0.0021	0.9973
P/T(3/1)	-ln(*C/C*_*0*_) = 0.0029*t*	0.0029	0.9948
P/T(4/1)	-ln(*C/C*_*0*_) = 0.0017*t*	0.0017	0.9944

**Figure 9:**
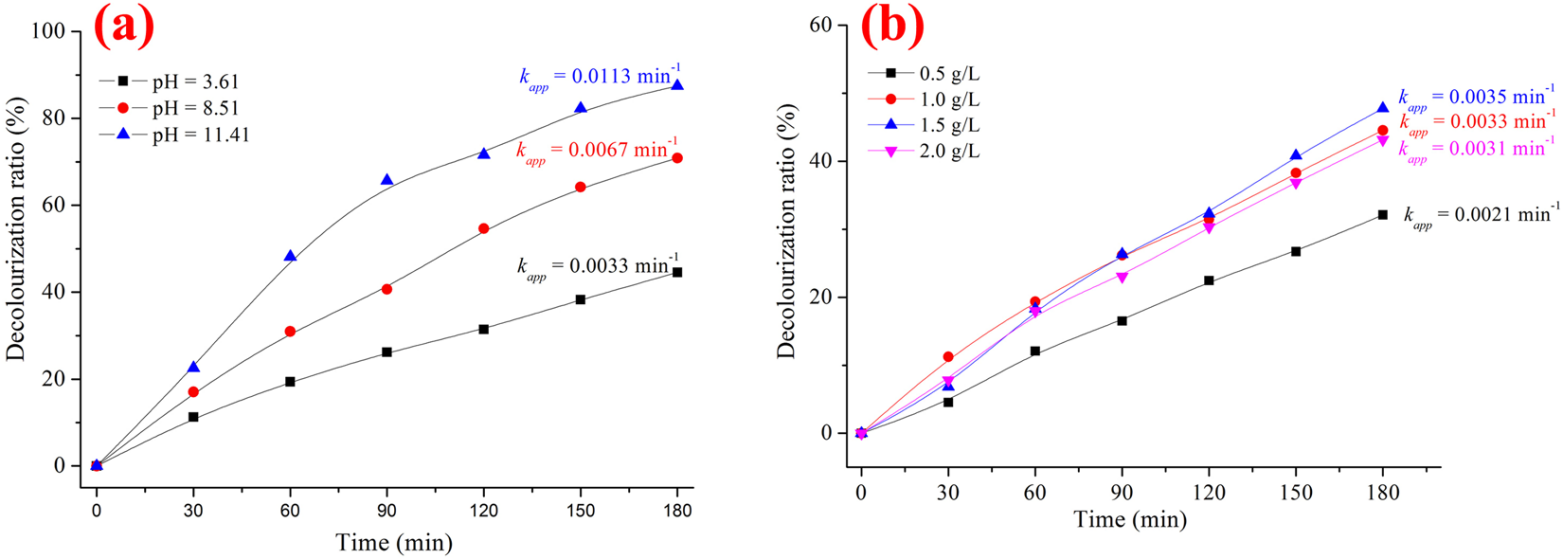
The decolorization ratio of MB under different (**a**) initial pH values and (**b**) concentrations of P/T(1/4).

To evaluate the photocatalytic activity of the P/T(1/4) nanocomposites with different initial pH values, the degradation of MB with an initial pH of 3.21, 8.07 and 11.35 is shown in Fig. 9(a). The results indicated that the photocatalytic activity of P/T(1/4) increased as the initial pH increased, and the first-order rate constant was 0.0033 min^−1^ for a pH of 3.61 and 0.0113 min^−1^ for a pH of 11.41. The higher photocatalytic activity at a higher initial pH was due to electrostatic adsorption between the photocatalyst and MB as well as the generation of ·OH^30^. On the one hand, a higher pH led to the photocatalyst with negative electricity, and MB was a typical cationic dye with a positive charge. Therefore, the electrostatic interaction was beneficial for enhancing the adsorptive property of P/T(1/4), which was also beneficial for the photocatalytic activity. On the other hand, a higher pH indicates rich OH^−^, and ·OH was generated by h^+^ and OH^−^. Therefore, a higher initial pH was beneficial for the generation of ·OH and enhanced photocatalytic activity.

To evaluate the photocatalytic activity of the P/T(1/4) nanocomposites with different photocatalyst concentrations, the degradation of MB with PoPD/TiO_2_ concentrations of 0.5 g/L, 1.0 g/L, 1.5 g/L, and 2.0 g/L is shown in Fig. 9(b). The results indicate that the photocatalytic activity of P/T(1/4) increased as the concentration of PoPD/TiO_2_ increased from 0.5 g/L (0.0021 min^−1^) to 1.5 g/L (0.0035 min^−1^), but it decreased as the concentration of PoPD/TiO_2_ increased from 1.5 g/L (0.0035 min^−1^) to 2.0 g/L (0.0031 min^−1^). This result indicates that the optimal concentration of the PoPD/TiO_2_ nanocomposite was 1.5 g/L. The photocatalytic activity with different PoPD/TiO_2_ concentrations was influenced by the absorption of visible light^31^. When the concentration of P/T(1/4) was low, the absorption of visible light increased as the P/T(1/4) concentration increased, which was beneficial for the generation of ROS. However, when the concentration of P/T(1/4) was high, the absorption of visible light decreased as the P/T(1/4) concentration increased owing to the obstructive effect from the excess photocatalyst, which was detrimental for the generation of ROS.

**Figure 10:**
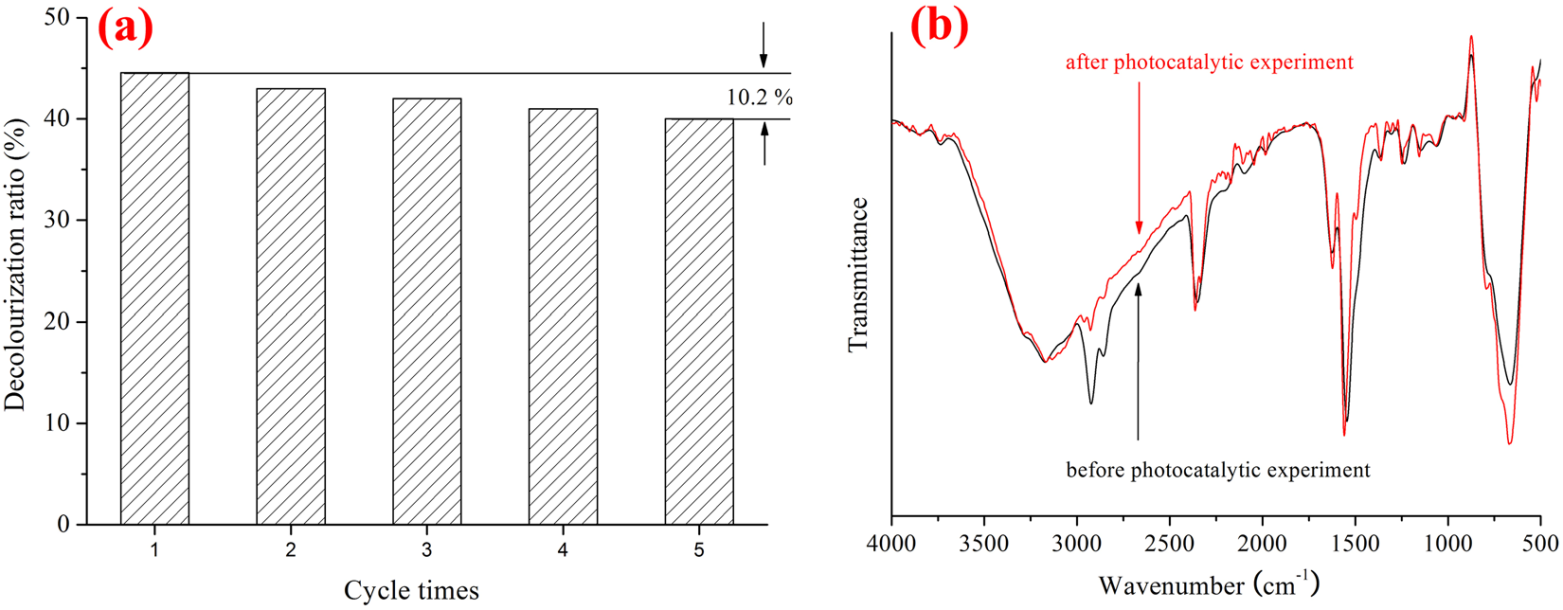
(**a**) The photocatalytic degradation rate of MB with the P/T(1/4) nanocomposite for different recycling times and (**b**) the FT-IR spectra of P/T(1/4) before and after the photocatalytic reaction.

In addition, some experiments were carried out to confirm the photocatalytic stability of the PoPD/TiO_2_ photocatalysts. It has been confirmed that PoPD/TiO_2_ exhibited good photocatalytic stability under irradiation conditions and continued to maintain its photocatalytic activity after five cycles, as shown in Fig. 10(a). The slight decrease in the photocatalytic activity during each cycle is due to a slight aggregation of the nanocomposites during the photocatalytic process. The FT-IR spectra of the PoPD/TiO_2_ nanocomposites before and after the reaction are shown in Fig. 10(b). The shape of the composite FT-IR spectrum after the photocatalytic experiment is similar to that of the particles prior to the experiment, which indicates that the structure of PoPD/TiO_2_ does not change during the photocatalytic process^32^. Therefore, the stability of the photocatalytic activity is dependent on the stability of the structure.

### Origin of oxidizing ability of PoPD/TiO_2_ nanocomposites on photocatalytic degradation of MB

**Figure 11:**
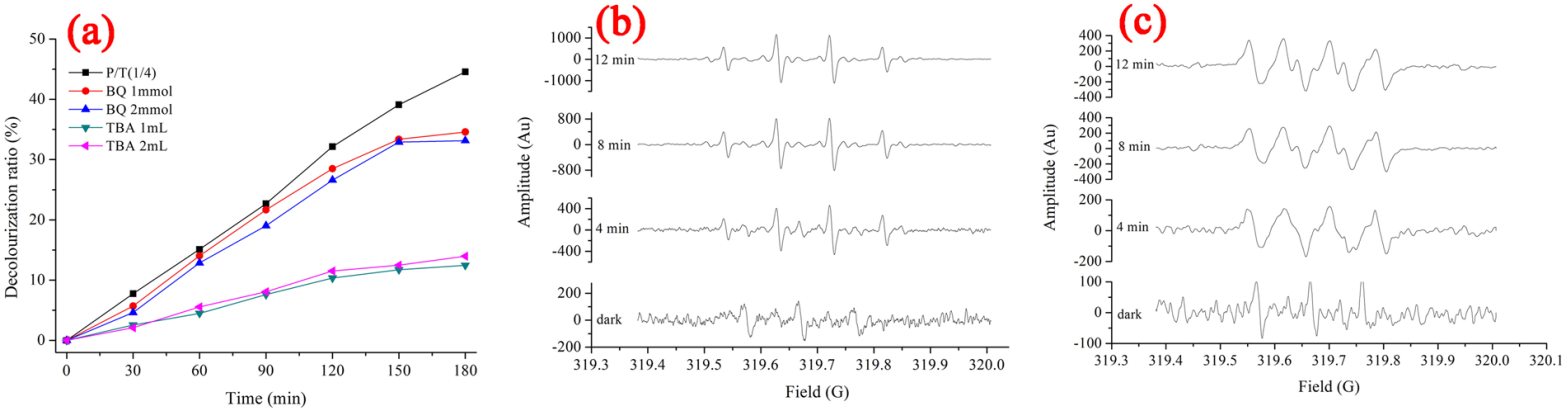
(**a**) Radical trapping experiments on photocatalytic degradation of MB with P/T(1/4) and ESR measurements of (**b**) ·OH and (**c**) ·O_2_−.

Some experiments were carried out to confirm the origin of oxidizing ability of PoPD/TiO_2_ nanocomposites on photocatalytic degradation of MB. On the one hand, to further evaluate the role of these active species such as ·OH and ·O_2_^−^, different types of active species scavengers are added in catalyst system. Figure 11a shows the photocatalytic activity of P/T(1/4) toward the degradation of MB under the different conditions. Without the addition of the scavengers, the photocatalytic decolourization ratio of MB is 44.5% (*k*_*app*_ = 0.0033 min^−1^) after 180 min of light irradiation. Since the PoPD itself is not only electron donors, but also the hole acceptors, a special kind of similar to the circulatory system between the PoPD and TiO_2_ will form when P/T is exposed to light. The benzoquinone (BQ) has the ability to trap ·O_2_^−^ by a simple electron transfer mechanism. The addition of BQ (1 mmol, 2 mmol) provokes partial inhibition of the MB degradation as shown in Fig. 11(a), and the related results of ESR are shown in Fig. 11(b) and 11(c). A combination of the results of ESR and the addition of BQ indicates that ·O_2_^−^ plays an important role in the photocatalytic process with decolourization ratio of MB decreasing from 44.5% to 34.6% (BQ 1 mmol) and 33.2% (BQ 2 mmol) and the first-order rate constant decreasing from 0.0033 min^−1^ to 0.0027 min^−1^ (BQ 1 mmol) and 0.0022 min^−1^ (BQ 2 mmol). Meanwhile, After 1 mL and 2 mL of tert-butyl alcohol (TBA) as a ·OH-scavenger are added into the reaction system, the rate for degradation of MB over P/T(1/4) is remarkably decreased. A combination of the results of ESR and the addition of TBA indicates that ·OH plays an more important role in the photocatalytic process with decolourization ratio of MB decreasing from 44.5% to 12.5% (TBA 1 mL) and 15.0% (TBA 2 mL) and the first-order rate constant decreasing from 0.0033 min^−1^ to 0.0008 min^−1^ (TBA 1 mL) and 0.0009 min^−1^ (TBA 2 mL).

### Photocatalytic mechanism and photocatalytic activity enhanced mechanism of the PoPD/TiO_2_ nanocomposites

**Figure 12:**
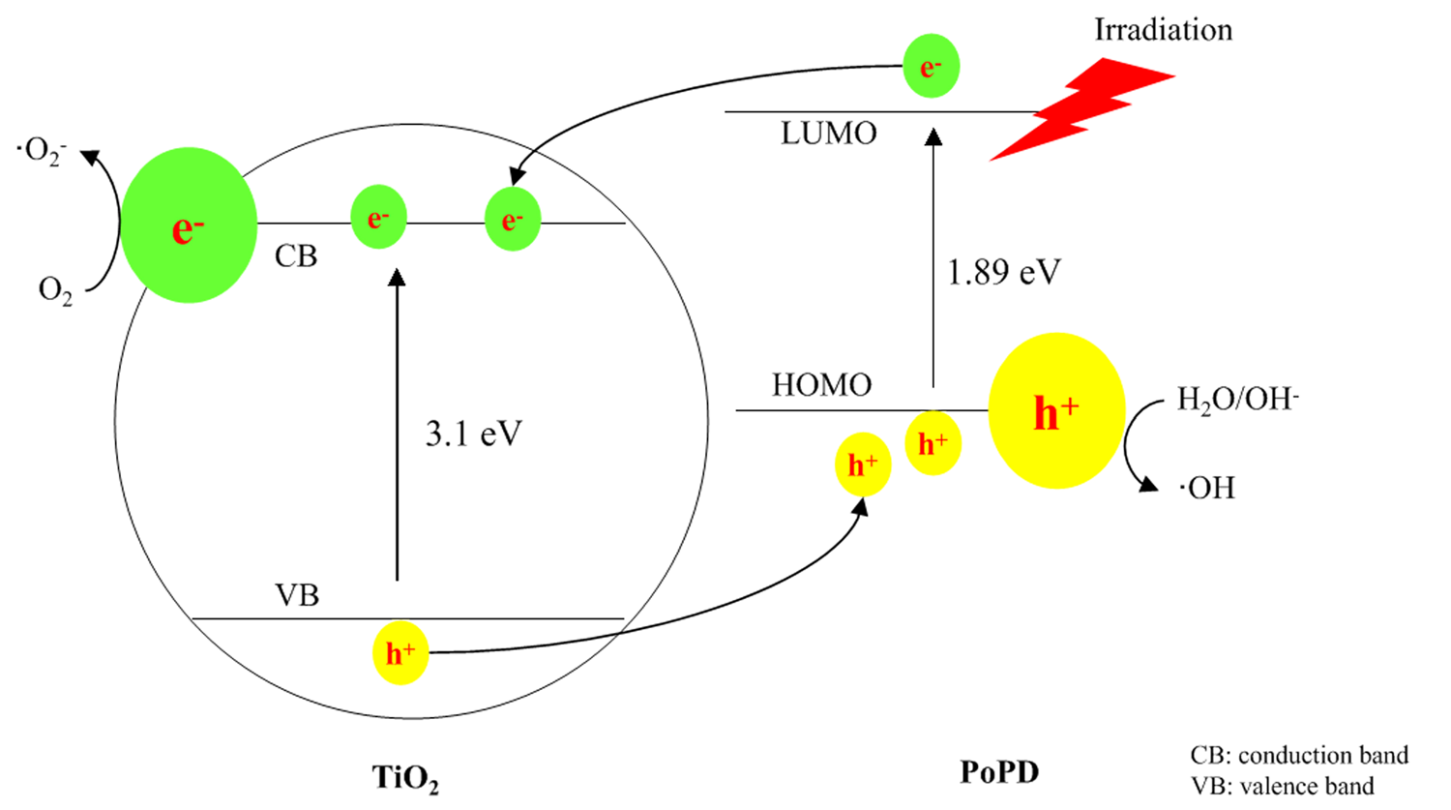
The photocatalytic mechanism of the PoPD/TiO_2_ nanocomposite.

The basic mechanism of PoPD/TiO_2_ nanocomposites was well established. TiO_2_ nanocomposite is irradiated with UV light to generate electron-hole pairs, which can react with water to yield hydroxyl and superoxide radicals, which oxidize and mineralize the organic molecules. However, the band gap of TiO_2_ is 3.11 eV, meaning that only UV light can excite the TiO_2_ nanocomposites to generate electron-hole pairs. One solution to overcome this shortcoming was to use a dye with a narrow band gap as a sensitizer to enhance the response of TiO_2_ to visible light. PoPD has a band gap of 1.89 eV, which is narrower than that of TiO_2_ (3.2 eV), showing strong absorption in the region of visible light. Therefore, PoPD can function as a photosensitizer for TiO_2_. When PoPD/TiO_2_ nanocomposites are illuminated under visible light, both TiO_2_ and PoPD absorb photons at their interface, where charge separation then occurs. This happens because the CB of TiO_2_ and the LUMO of PoPD are well matched for charge transfer. Electrons generated by conducting PoPD can be transferred to the conduction band of TiO_2_, enhancing charge separation and promoting the photocatalytic ability of the P/T(1/4). The synergetic effect between PoPD and TiO_2_ on the photocatalytic degradation of MB clearly existed in not all the PoPD/TiO_2_ nanocomposites^33^. An optimum synergetic effect was found in P/T(1/4). The effect of PoPD on the activity of the P/T(1/4) nanocomposite can be explained by its action as photosensitizer in Fig. 12. The doped PoPD semiconductive material can absorb visible light irradiation and transfer the photogenerated electron (e^−^) into the conduction band (CB) of TiO_2_^34^. Simultaneously, a positively charged hole (h^+^) might be formed from the transfer of an electron from the TiO_2_ valence band (VB) to PoPD^35^. This electron transfer between PoPD and the TiO_2_ semiconductor, as well as the enhanced photocatalytic activity of the composites, has been experimentally observed in other systems^36^. The free electrons reacted with O_2_ to produce superoxide radical (·O_2_^−^), and the holes (h^+^) reacted with OH^−^ and H_2_O to produce a hydroxyl radical (·OH). The MB solution was degraded by the reactive oxygen species (ROS) (e.g., ·O_2_^−^ and ·OH).

The photocatalytic mechanism of PoPD/TiO_2_ is as follows^37^:

1 $${\mathrm{PoPD}/\mathrm{TiO}}_{{\rm{2}}}+{\rm{hv}}\to {{\rm{PoPD}}}^{+}{/\mathrm{TiO}}_{{\rm{2}}}+{{\rm{e}}}^{-}$$

2 $${{\rm{e}}}^{-}+{{\rm{O}}}_{{\rm{2}}}\to \cdot {{{\rm{O}}}_{{\rm{2}}}}^{-}$$

3 $${{\rm{PoPD}}}^{+}/\mathrm{TiO2}\to \mathrm{PoPD}/\mathrm{TiO2}+{{\rm{h}}}^{+}$$

4 $${{\rm{h}}}^{+}+{{\rm{H}}}_{{\rm{2}}}{O/\mathrm{OH}}^{-}\to \cdot {\rm{OH}}$$

5 $${\rm{MB}}+{\rm{ROS}}\to {{\rm{CO}}}_{{\rm{2}}}+{{\rm{H}}}_{{\rm{2}}}{\rm{O}}$$

Although the photocatalytic activity of the photocatalyst was influenced by many factors, two key factors were identified based on the photocatalytic mechanism. In the primary reaction process, TiO_2_ is excited under light irradiation to generate electron-hole pairs. In addition, in the secondary reaction process, the ROS is produced to degrade organic pollutants. Therefore, the two key factors include solar absorption and charge separation.

For PoPD/TiO_2_, because PoPD is a photosensitizer with a narrow level spacing, PoPD/TiO_2_ inserts the energy level of PoPD into the energy level of TiO_2_ to enhance its response to visible light. On the other hand, owing to the synergetic effect of the well-matched energy levels of TiO_2_ and PoPD, PoPD/TiO_2_ hinders the recombination of the hole and electron to generate more ROS that degrade MB. Therefore, we can explain that the photocatalytic activity enhanced mechanism is based on a photosensitization effect as well as a synergetic effect^38, 39^.

### Photosensitization effect to enhance the response to visible light

For a crystalline semiconductor, the optical absorption near the band edge follows the Kubelka-Munk function^40^ (see Supporting Information Equation 1), and the results are shown in Fig. 7. The band gap energies for TiO_2_, PoPD and PoPD/TiO_2_ were determined to be 3.10 eV, 1.89 eV and 2.45 eV, respectively, which indicated that PoPD/TiO_2_ is a better photocatalyst than the unmodified TiO_2_ owing to PoPD being a photosensitizer. The excitation between the HOMO and LUMO in PoPD is much lower because the benzene rings are conjugated through an imine linkage and the sulphonyl is an electron-withdrawing group. The experimental absorption spectrum for PoPD indicates a band gap of 1.89 eV, which is interpreted as excitations to the polaron band. In addition, PoPD is an efficient electron donor and hole transporter upon light excitation^41^. In the combined system with PoPD and TiO_2_, the response of light expands from 400 nm (UV) to 506 nm (visible light), which were obtained from the wavelength values corresponding to the intersection point of the vertical and horizontal portions of the spectra using *hc/λ* = *E*_*g*_, where *E*_*g*_ is the band gap energy, *h* is the Planck’s constant, *c* is the light velocity (m/s), and *λ* is the wavelength (nm). Therefore, because of the photosensitization effect of PoPD, the PoPD/TiO_2_ nanocomposites exhibit a stronger response to visible light, and the photocatalytic activity of PoPD/TiO_2_ was enhanced owing to the enhanced response to visible light.

### Synergetic effect to enhance the generation of ROS

The synergetic effect between TiO_2_ and PoPD on the photocatalytic degradation of MB exists in PoPD/TiO_2_ because of the well-matched energy levels, and the optimum synergetic effect was observed for P/T(1/4). The energy levels of TiO_2_ and PoPD are as follows: *E*_*(LUMO)*_ > *E*_*(CB)*_ > *E*_*(HOMO)*_ > *E*_*(VB)*_^42^. Under irradiation, the electrons are excited from the HOMO to the LUMO to the CB. Then, the holes transfer from the VB to the HOMO owing to PoPD being a hole transporter. Therefore, electrons and holes gather in the CB of TiO_2_ and the HOMO of PoPD, respectively. This behavior is favorable for enhancing the quantum efficiency as the separation efficiency between the hole and electron increases. The synergetic factor (*f*) can be calculated based on the apparent first-order kinetics (see Supporting Information Equation 2) as $f=\frac{{k}_{C/T}}{{k}_{T}}$, where *k*_*C/T*_ is the first-order rate constant of PoPD/TiO_2_ and *k*_*T*_ is the first-order rate constant of TiO_2_^43^. Based on the apparent first-order kinetic constants of the degradation of MB from Table 2, the synergetic factors of the PoPD/TiO_2_ nanocomposites are 0.86 for P/T(1/6), 1.10 for P/T(1/5), 1.57 for P/T(1/4), 0.95 for P/T(1/2), 0.38 for P/T(1/1), 1.00 for P/T(2/1), 1.38 for P/T(3/1), and 0.81 for P/T(4/1). Therefore, the photocatalytic activity of PoPD/TiO_2_ was enhanced by increasing the quantum efficiency.

**Figure 13:**
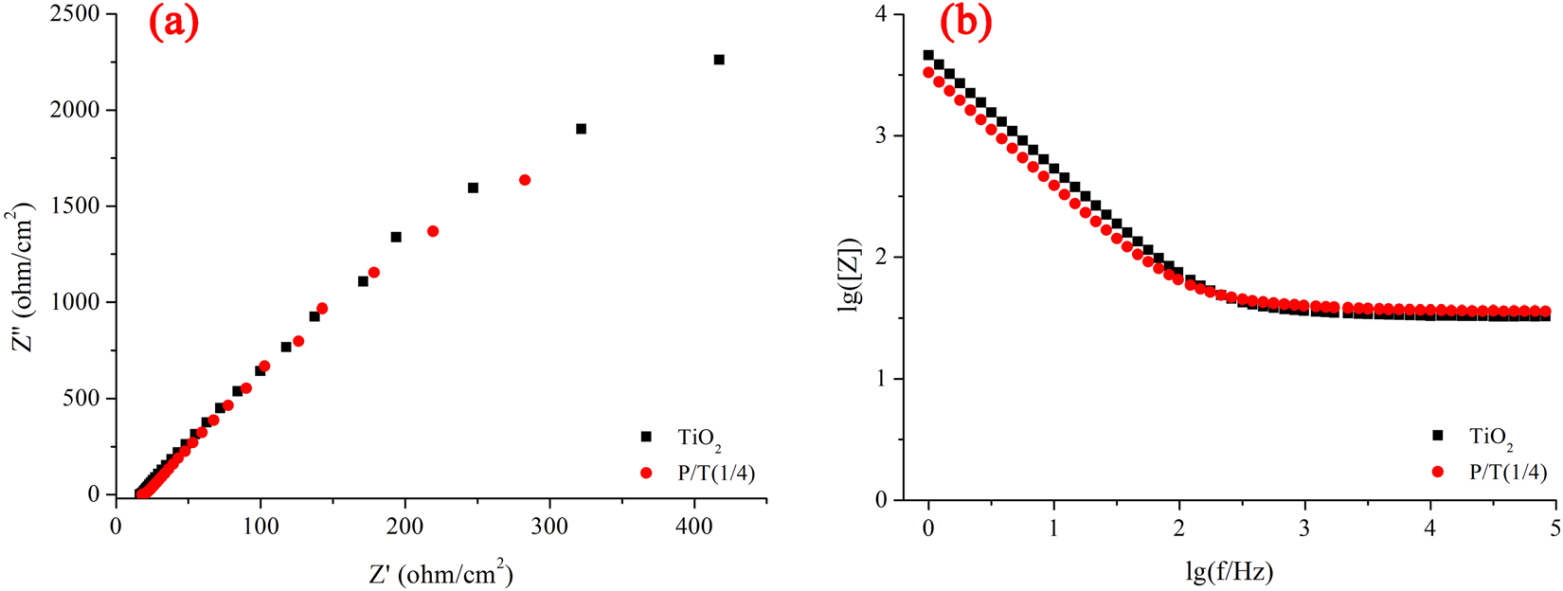
Electrochemical impedance spectroscopy of samples. (a and b represent the Nyquist plot and Bode plot, respectively).

Moreover, electrochemical impedance spectroscopy (EIS) as a common electrochemical method has been widely used in evaluating the interface charge transfer efficiency and separation of photogenerated electron-hole pairs over the photocatalyst. As shown in Fig. 13(a) and 13(b), it is so clearly that the radius of the arc on the EIS Nynquist plot of P/T(1/4) is smaller than that of the TiO_2_, which reflects that P/T(1/4) possesses the faster interfacial charge transfer, and the impedance on the EIS Bode plot of P/T(1/4) is smaller than that of the TiO_2_, which reflects that P/T(1/4) possesses the higher photocatalytic activity. The results of EIS test is well correspond to that of photocatalytic experiments. According to the EIS, it is indicated that the presence of PoPD in the P/T(1/4) nanocomposites is capable of improving separation efficiency and effectively inhibit the electron-hole pair recombination.

## Conclusions

PoPD/TiO_2_ nanocomposites were synthesized via an ‘*in situ*’ oxidative polymerization method. The modified photocatalysts were characterized by BET, XRD, TEM, FT-IR, TGA, XPS, EA and UV-Vis DRS. The results indicated that the PoPD exists on the surface of TiO_2_, the presence of PoPD does not impact on the lattice structure and grain size of TiO_2_, and the presence of PoPD enhaces the visible light response. The photocatalytic degradation of methylene blue (MB) was chosen as a model reaction to evaluate the photocatalytic activities of TiO_2_ and PoPD/TiO_2_. The results indicated that PoPD/TiO_2_ nanocomposites exhibited good photocatalytic activity (apparent first-order rate constants of 0.0021 min^−1^ for TiO_2_ and 0.0033 min^−1^ for P/T(1/4)) and stability (recycled 5 times, 15 hours of operation). The photocatalytic activity of PoPD/TiO_2_ was influenced by the initial pH and concentration of the photocatalyst. Based on radical trapping experiments and ESR measurements, the origin of oxidizing ability of PoPD/TiO_2_ nanocomposites on photocatalytic degradation of MB was proposed, which taking into account of ·OH was the first important ROS and ·O_2_^−^ was the second important ROS. The photocatalytic activity enhanced mechanism has been proposed, taking into account the photosensitization effect (UV-Vis DRS) and synergetic effect of TiO_2_ with PoPD (Synergetic Factor and EIS).

## Electronic supplementary material

Supplementary Material
