# Supplementary material for: Highly-efficient photocatalytic degradation of methylene blue by PoPD-modified TiO2 nanocomposites due to photosensitization-synergetic effect of TiO2 with PoPD
Source: Sci Rep. 2017 Jun 21;7:3973. doi: 10.1038/s41598-017-04398-x (PMC5479840; doi:10.1038/s41598-017-04398-x)
Supplement: Supplementary file 1 — Supplementary Material [file 41598_2017_4398_MOESM1_ESM.doc]

Supplementary Material

**Highly-efficient photocatalytic degradation of methylene blue by PoPD-modified TiO_2_ nanocomposites due to photosensitization-synergetic effect of TiO_2_ with PoPD**

Chuanxi Yang^a,b^, Wenping Dong^c^, Guanwei Cui^d^, Yingqiang Zhao^d^, Xifeng Shi^d^, Xinyuan Xia^d^, Bo Tang^* d^, and Weiliang Wang^* a^

^a^ College of Geography and Environment, Shandong Normal University, Jinan 250014, P. R. China

^b^ College of Resources and Environmental Sciences, China Agricultural University, Beijing 100193, P. R. China

^c^ Shandong Academy of Environmental Science and Environmental Engineering Co, Ltd, Jinan 250013, P. R. China

^d^ College of Chemistry, Chemical Engineering and Materials Science, Collaborative

Innovation Center of Functionalized Probes for Chemical Imaging in Universities

of Shandong, Key Laboratory of Molecular and Nano Probes, Ministry of

Education, Shandong Provincial Key Laboratory of Clean Production of Fine

Chemicals, Shandong Normal University, Jinan 250014, P. R. China

* Corresponding author: Fax: (+86) 531-8618-0017; (+86) 531-8618-2550

E-mail address: tangb@sdnu.edu.cn; sdqcsdnu@163.com

**Equation 1**: The calculation of the band gap of TiO_2_, PoPD and PoPD/TiO_2_ nanocomposites based on the Kubelka-Munk function.

$F\left(R\right)E=A\left(E-E_{g}\right)^{n/2}$ (Equation 1a)

where *F(R)*, *E*, *A*, *n* and *E_g_* are the diffuse absorption coefficient, photon energy, proportionality constant, an integer (n=1, 2, 4, and 6), and band gap, respectively. For TiO_2_, PoPD and PoPD/TiO_2_, the relationship between the diffuse absorption coefficient and the band gap energy can be described by Equation 1b.

$\left(F\left(R\right)E\right)^{1/2}=A\left(E-E_{g}\right)$ (Equation 1b)

For Equation 1b, *(F(R)E)^1/2^* has a linear relationship with *E*. For the samples, the value of *n* was determined to be 4. Therefore, the optical transitions of the crystal were indirectly forbidden. The band gap energies for TiO_2_, PoPD and PoPD/TiO_2_ were determined to be 3.10 eV, 1.89 eV and 2.45 eV, respectively, which indicated that PoPD/TiO_2_ is a better photocatalyst than the unmodified TiO_2_ owing to PoPD being a photosensitizer.

[**Equation**](mailto:sdqcsdnu@163.comEquation) **2**: The calculation of synergetic factor *f* of PoPD/TiO_2_ based on the apparent first-order kinetics.

$-\frac{dR}{dt}=k_{C/T}\left[C_{t}\right]=k_{C}\left[C_{t}\right]+k{}_{T}\left[C_{t}\right]+k_{C-T}\left[C_{t}\right]$ (Equation 2a)

where *k_C/T_* is the first-order rate constant of PoPD/TiO_2_, *k_C_* is the first-order rate constant of PoPD, *k_T_* is the first-order rate constant of TiO_2_, and *[C_t_]* is the concentration of MB at the same moment. Therefore, the photocatalytic degradation of MB can be identified as the degradation of TiO_2_, PoPD, and the synergetic effect between PoPD and TiO_2_. The synergetic factor can be calculated via the following Equation 2b:

$f=\frac{k_{C/T}}{k_{C}+k_{T}}$ (Equation 2b)

Because the mass of TiO_2_ is much larger than that of PoPD, and PoPD is used as a modifier with no obvious photocatalytic activity, the synergetic factor is calculated via the following Equation 2c:

$f=\frac{k_{C/T}}{k_{T}}$ (Equation 2c)

Based on the apparent first-order kinetic constants of the degradation of MB, the synergetic factors of the PoPD/TiO_2_ nanocomposites are 0.86 for P/T(1/6), 1.10 for P/T(1/5), 1.57 for P/T(1/4), 0.95 for P/T(1/2), 0.38 for P/T(1/1), 1.00 for P/T(2/1), 1.38 for P/T(3/1), and 0.81 for P/T(4/1). The results indicated that not all of the PoPD/TiO_2_ nanocomposites showed higher photocatalytic activity than TiO_2_, but an optimizing condition was obtained with an oPD to TiO_2_ initial molar ratio of 1/4.
